# Multi-Temporal Change Detection Analysis of Vertical Sprawl over Limassol City Centre and Amathus Archaeological Site in Cyprus during 2015–2020 Using the Sentinel-1 Sensor and the Google Earth Engine Platform

**DOI:** 10.3390/s21051884

**Published:** 2021-03-08

**Authors:** Athos Agapiou

**Affiliations:** 1 Department of Civil Engineering and Geomatics, Faculty of Engineering and Technology, Cyprus University of Technology, Saripolou 2-8, 3036 Limassol, Cyprus; athos.agapiou@cut.ac.cy; Tel.: +357-25-002471; 2 Eratosthenes Centre of Excellence, Saripolou 2-8, 3036 Limassol, Cyprus

**Keywords:** change detection, multi-temporal analysis, heritage management, Sentinel-1, Google Earth Engine, remote sensing archaeology, urban sprawl, vertical sprawl

## Abstract

Urban sprawl can negatively impact the archaeological record of an area. In order to study the urbanisation process and its patterns, satellite images were used in the past to identify land-use changes and detect individual buildings and constructions. However, this approach involves the acquisition of high-resolution satellite images, the cost of which is increases according to the size of the area under study, as well as the time interval of the analysis. In this paper, we implemented a quick, automatic and low-cost exploration of large areas, for addressing this purpose, aiming to provide at a medium resolution of an overview of the landscape changes. This study focuses on using radar Sentinel-1 images to monitor and detect multi-temporal changes during the period 2015–2020 in Limassol, Cyprus. In addition, the big data cloud platform, Google Earth Engine, was used to process the data. Three different change detection methods were implemented in this platform as follow: (a) vertical transmit, vertical receive (VV) and vertical transmit, horizontal receive (VH) polarisations pseudo-colour composites; (b) the Rapid and Easy Change Detection in Radar Time-Series by Variation Coefficient (REACTIV) Google Earth Engine algorithm; and (c) a multi-temporal Wishart-based change detection algorithm. The overall findings are presented for the wider area of the Limassol city, with special focus on the archaeological site of “Amathus” and the city centre of Limassol. For validation purposes, satellite images from the multi-temporal archive from the Google Earth platform were used. The methods mentioned above were able to capture the urbanization process of the city that has been initiated during this period due to recent large construction projects.

## 1. Introduction

Cultural heritage monuments and landscapes are threatened by various anthropogenic and natural hazards [1,2,3], including urbanization. Despite the urbanisation process being considered a relatively slow process compared to earthquakes and other hazard events that can have an immediate impact on cultural heritage sites, several studies indicated that urban sprawl can similarly impact the archaeological record of an area in the long term [4,5]. In addition, as [6] argue, new global challenges are expected in terms of balancing cultural heritage conservation and addressing the requirements of the urbanization process.

However, the difficulty to assess the impact of the urban sprawl is related on one hand to the nature of the hazard, which is a result of a complex set of interrelated socioeconomic and cultural forces, while on other hand, the urbanization process which can last for several years. Based on the outcomes of the “Copernicus Services in Support to Cultural Heritage” report [7], nine domains have been identified as high-level user needs. Among them, three domains include the (a) monitoring of the evolution of the environment of the cultural heritage site; (b) the mapping of the cultural landscape of the site and the identification of specific risk; and (c) the observation of changes of a cultural heritage site. Therefore, in order that local stakeholders understand the impact of the urbanization, multi-temporal analyses from available data sources are necessary. In this respect, earth observation sensors and satellite images are considered fundamental to tackle this issue.

The technological improvements observed in recent years in the domain of earth observation and big data image analysis have benefited a wide range of remote sensing applications allowing time-series analysis. A scientific literature overview indicates the continuous increase in studies dealing with new satellite sensors and big-data analysis using cloud-based platforms [8,9,10,11].

During the last decade, the European Copernicus Programme [12] has been considered a milestone of the Earth Observation’s history. During this decade (2010–2020) and under the Copernicus Programme, the Sentinel-1A and -1B radar sensors have been launched and set into orbit, providing medium spatial resolution radar images at a high-temporal revisiting time [13]. In parallel, national-driven, and commercial big-data cloud platforms have been released, ready to explore the available freely distributed amount of satellite imageries [14,15]. For several research themes, the processing of multi-temporal Sentinel-1 datasets through cloud-based platforms has been well introduced in the recent past [16,17]. Nevertheless, in other themes, the benefit of this technological shift is still under-examined.

Specifically for heritage management, while earth observation has been well introduced in the literature, the majority of these studies are focused on the exploitation of a limited number of high-resolution optical sensors [18,19,20,21] with some noticeable exceptions [22,23]. Even fewer studies are found in the literature to be dealing with the use of radar sensors at a medium resolution [24,25]. Indeed, the use of the Copernicus Sentinel-1 radar sensor [26] is still limited for heritage management applications, with exceptions in the application of interferometric synthetic-aperture radar (In-SAR) analysis [27,28]. This is well reported in [29] where the coarse spatial resolution of the radar data and the signal interference limits the potential use of the Sentinel-1 sensor.

Consequently, in contrast to the optical processing chain, radar sensor big data multi-temporal applications are still rare for heritage management. Therefore, there is a need to evaluate the utility of the freely distributed radar images provided by the Sentinel-1 sensors, in the context of heritage management over extensive areas.

Here, we present a multi-series analysis of radar images, through big data cloud platform to map landscape changes in a quick and automatic procedure, thus allowing local stakeholders to visualize the impact and the changes related to the urban sprawl. While, due to the resolution of the images, individual buildings cannot be detected, it is important to have such low-cost exploration tools for a quick assessment of the landscape changes. For this study’s aims, available Sentinel-1 images from Google Earth Engine were processed, covering a period of five-years (2015–2020) and selecting the city of Limassol, in Cyprus, as a case study area. The overall results were then compared with high-resolution Google Earth historical images.

## 2. Case Study

In Cyprus, the urbanisation process has been documented in the past though remote sensing sensors. For instance, in the Paphos District, an increase of 300% of the urban footprint was mapped after analysing Landsat images through supervised classification analysis over 30 years (1980–2010) [30]. This urban expansion was mainly observed in the western part of the Paphos city; however, the rest of the island cities have shown a similar trend. During this period, archaeological rescue excavations revealed significant archaeological records, like the Hellenistic and Roman tombs in Paphos [31,32], underlining the numerous subsurface wealth of archaeological findings of the island.

In recent years, urbanisation and development patterns have changed on the island. After the economic crisis that hit Cyprus in 2012, the construction industry was considered a central pillar for its future economic growth. Since 2015 and based on the statistics [33], land development was supported through large constructions, the so-called high towers, and other development projects beyond the human scale. This is a phenomenon known in the literature as vertical sprawl.

While these developments were a phenomenon that has been reported during the period 2015–2020 all over the island, Limassol city was by far the most affected. More than 30 high towers have been constructed or are currently under construction within the city, and near the Amathus archaeological site.

Despite the fact that this new type of construction project (i.e., high towers) can be considered as a sustainable solution, since it can open up the surrounding rural space [34], in cities like Limassol, the new constructions are built on the few empty plots of the city remaining. Sometimes, smaller older buildings were demolished to secure free space. Other areas in the suburbs of the city near archaeological sites have been selected for these construction projects. 

**Figure 1:**
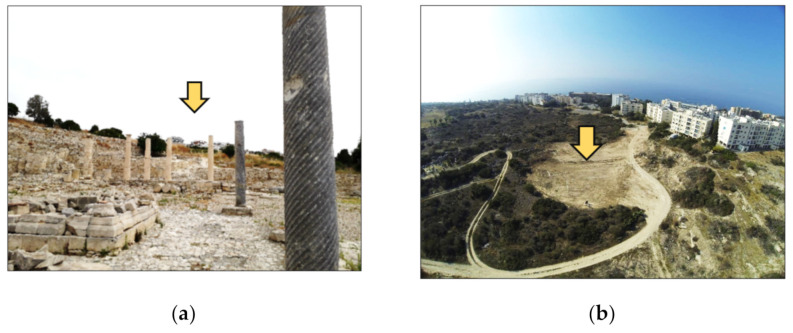
(**a**) New constructions at the eastern part of the Amathus archaeological site indicated with an arrow. (**b**) New areas developed, in the eastern part of the Amathus site, whereas geophysical prospection surveys have been carried out by the Laboratory of Geophysics—Satellite Remote Sensing and Archaeoenvironment, Institute for Mediterranean Studies (IMS), Foundation for Research and Technology Hellas (FORTH) (photos taken in 2015).

During this new construction wave, archaeologists have been struggling against time, to perform rescue excavations over large areas. Archaeological findings are used to support local authorities’ arguments, in modifications and adjustments of the architectural designs of the high towers. Indeed, non-systematic archaeological excavations have been performed by the local stakeholders (Department of Antiquity of Cyprus) to rescue and record the archaeological findings. In this attempt, the geophysical prospection and archaeological investigations have been performed to support local stakeholders and record the archaeological landscape (Figure 1).

**Figure 2:**
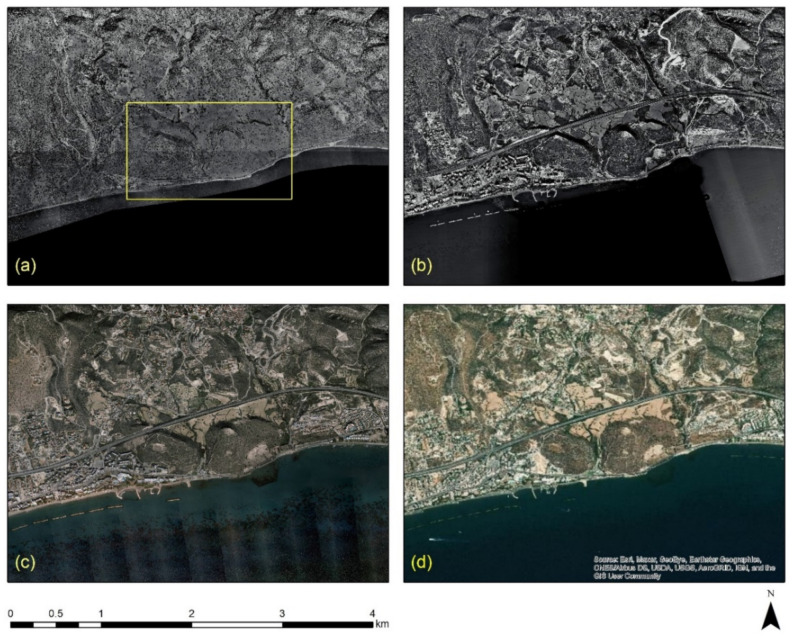
A diachronic overview of Limassol city, Cyprus, using archive aerial and satellite images. The archaeological site of Amathus is shown with a yellow rectangle: (**a**) orthophoto image of 1963; (**b**) orthophoto image of 1993; (**c**) orthophoto image of 2014; and (**d**) a recent satellite image from ArcGIS online database (source of aerial orthophotos: Department of Land and Surveyors).

A historical overview of the urban expansion of the Limassol city is shown in Figure 2. Available aerial images (orthophotos) distributed by the Department of Land and Surveyors, Cyprus [35], have been imported as a Web Map Service (WMS) in a Geographical Information System (GIS) environment (ArcGIS v.10.6). Figure 2a shows the orthophoto of 1963 over the Amathus archaeological site (with a yellow rectangle) and its surrounding area; Figure 2b shows the same area from the aerial photo of 1993; Figure 2c the area as depicted from the orthophoto of 2014 and Figure 2d a recent satellite image from the ArcGIS online database. As shown in Figure 2, urban areas are observed in the western and eastern parts of the archaeological site, while new construction developments have also been mapped in the northern part of the site.

## 3. Materials and Methods

In this study, the Google Earth Engine cloud platform was used [14]. The platform allows researchers to access various satellite-based products and satellite images, supporting large-scale remote sensing applications. As the specific platform enables the free programmatic access to a list of satellite data, with continual updates, several researchers have released in the recent past new algorithms exploiting the benefits of this platform [36].

The latest can assist other researchers with limited background programming skills to take into advantage of the Google Earth Engine platform and satellite data availability by either implementing or modifying existing algorithms. In this study, we focused on three different change detection methods using the free and open distributing Copernicus Sentinel-1 images. Sentinel-1 sensors provide dual-polarisation C-band Synthetic Aperture Radar (SAR) images. Sentinel-1 collection at Google Earth Engine includes Ground Range Detected (GRD) scenes that are processed using the Sentinel-1 Toolbox to generate a calibrated, ortho-corrected products [37].

**Figure 3:**
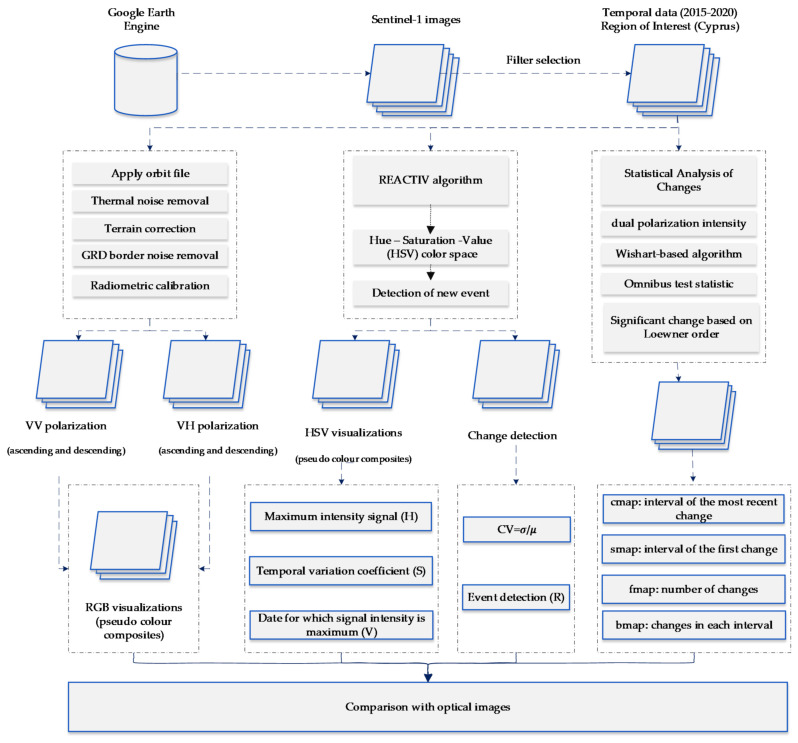
The overall methodology implemented in the current study, where: REACTIV corresponds to the “Rapid and Easy Change Detection in Radar Time-Series by Variation Coefficient” algorithm, VV to the “vertical transmit, vertical receive polarisation, VH to the “vertical transmit, horizontal receive polarisation”, GRD to the “Ground Range Detected” and CV to the “coefficient variation”.

Figure 3 shows the overall methodology implemented here. At first, Sentinel-1 GRD data were selected and then filtered to cover 2015–2020 over the Limassol city. It should be mentioned that during this period, Sentinel-1 has become available and the large constructions have been populated over the Limassol city.

In the beginning, a red–green–blue (RGB) pseudo colour visualisation was produced using the VV (vertical transmit, vertical receive) and the VH (vertical transmit, horizontal receive) polarisation images based on the multi-temporal Sentinel-1 dataset. All images are radiometrically corrected, as shown in Figure 3 (left). The visualisation products were used as a first proxy to detect new areas of changes.

At a second step, the so-called Rapid and Easy Change Detection in Radar Time-Series by Variation Coefficient (REACTIV) algorithm [38,39] was implemented at the Google Earth Engine. The REACTIV script can be found in [40]. The specific algorithm visualises a stack of multi-temporal Sentinel-1 SAR images based on the hue–saturation–value (HSV) colour transformation [see more on 41]. The VV and VH Sentinel-1 were used for this purpose in both ascending and descending orbits. The hue component encodes the dating information of the event (change). For this purpose, the time index of maximum signal value for the pixel across all polarisations is estimated. The time difference between the maximal time index and the time index (event) is calculated divided by the maximal and minimal time index difference. Then, the results are rescaled between 0 and 1. The saturation component indicates the changing intensity. Significant changes are highlighted with saturated colour, while low saturation indicates areas with no or minimum observation period changes. Last, the value component represents the input signal’s maximum value over both Sentinel-1 polarisations (VV and VH). As argued by [41], the value component does not provide sufficient details for change; however, it is used to improve the overall visualisation.

Finally, at the third step, a change detection statistical analysis of the multi-temporal Sentinel-1 series was implemented. Measuring the bitemporal changes between the all-possible pairs of the Sentinel-1 images would eventually provide a high false positive rate. For instance, for the year 2018 (2018-01-01 until 2018-12-31), 56 Sentinel-1 images were accessible through the Google Earth Engine. The false positive error can be up to 42%, for a given a = 0.01, considering that these individual pairs are statistically independent (false positive error = 1 − (1 − 0.01)^56 − 1^). For this reason, Ref. [42] have proposed a multi-temporal Wishart-based change detection algorithm. Three new change detection indicators can be estimated after the application of this algorithm: the so-called “cmap” that indicates the interval (time) of the most recent change, the “smap” that implies for the interval of the first change, and the “fmap” that shows the number of changes during the observation period. In addition, changes for each interval can be generated (“bmap”).

The overall results from these three different techniques are then compared with multi-temporal high-resolution RGB satellite data from the Google Earth platform. Significant changes are mapped in the ArcGIS (v.10.6) Geographical Information System (GIS) software. Further cross-evaluation of the above products was also carried out, including masking non-urban areas from classified Sentinel-2 image.

## 4. Results

This section presents the results from the overall analysis of the Sentinel-1 datasets using the three change detection techniques as described above and shown in Figure 2.

### 4.1. Visual Interpretation of VV and VH Polarisations

**Figure 4:**
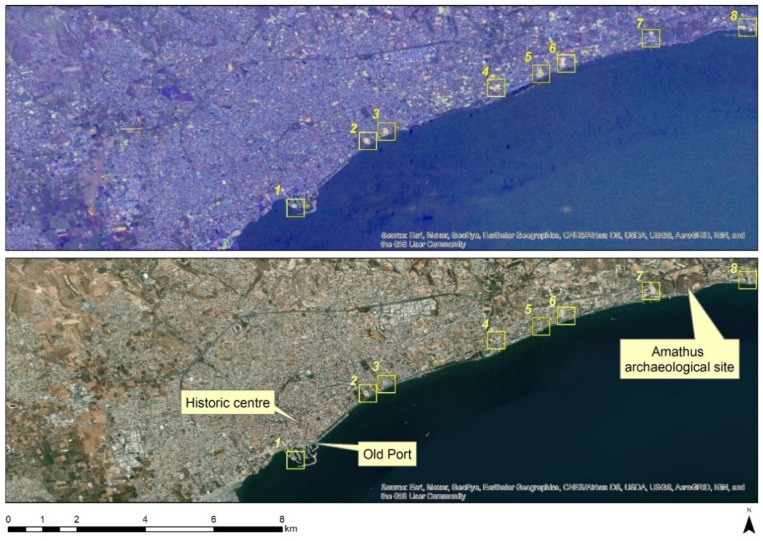
Top: VV and VH polarisation visualisation change detection during the period 2015–2020 over Limassol city. Bright colours indicate areas of changes during this period. Bottom: The same are using as a background a high-resolution satellite image (source: ArcGIS Basemap). Examples from these changes are provided in Table 1 for the selected areas indicated with a yellow square (no. 1–no. 9).

Initially, a visual interpretation of VV and the VH polarisation of the Sentinel-1 images over the area of Limassol was performed. Both ascending and descending orbits were used to create a pseudo-colour composite that can enhance the urban footprint as described in [43]. An RGB pseudo colour composite for the years 2015–2020 was created as follows: red for the VV polarisation, green for the VH polarisation, and blue for the ration of the VV/VH polarisations. In addition, the difference between the annual datasets of 2020 and 2015 were estimated. The finals results are shown in Figure 4.

Areas indicated in bright colour, in Figure 4, suggest a significant change in the polarisation signal during the period 2015–2020. Changes are observed within the Limassol city and its surrounding area. While on the western outskirts of Limassol these changes are fewer, a significant number of changes can be observed both in the centre of the city (Figure 4) and at the Amathus archaeological site (see yellow squares no. 7 and no. 8, Figure 4). Most of the land-use changes are also recorded along the seafront of the city, starting from the so-called “Old-Port” of the city (Figure 4) until the eastern part of the city, and near to the Amathus site (Figure 4).

A closer look at selected areas located in the broader area of Limassol city indicated in Figure 4 with yellow squares (no. 1–no. 8), is provided in Table 1. Screenshots from the Google Earth digital globe were exported, using: (a) the most recent satellite RGB image of the area (date of acquisition: 12 September 2020) and (b) the satellite image during the observation time (date of acquisition: 24 January 2015). The eight examples provided in Table 1, are located along the coastline of the Limassol city, starting from the “Old Port” (see Figure 4) towards the eastern part of the Amathus archaeological site (see Figure 4).

The first example (no. 1, Table 1) refers to new constructions that have been developed in the “Marina of Limassol” area, a multi-million development project that still runs. New sky-towers and very high buildings can be seen in the rest of the selected areas (no. 2 to no. 8, Table 1). The developers have taken advantage of large empty plots for their construction and development plans. After the rescue excavations carried out by the Department of Antiquities of Cyprus, archaeological findings have been revealed in the cases of no. 7 and no. 8, which are in the western and the eastern parts of the Amathus archaeological site.

### 4.2. HSV Colour Transformation

**Figure 5:**
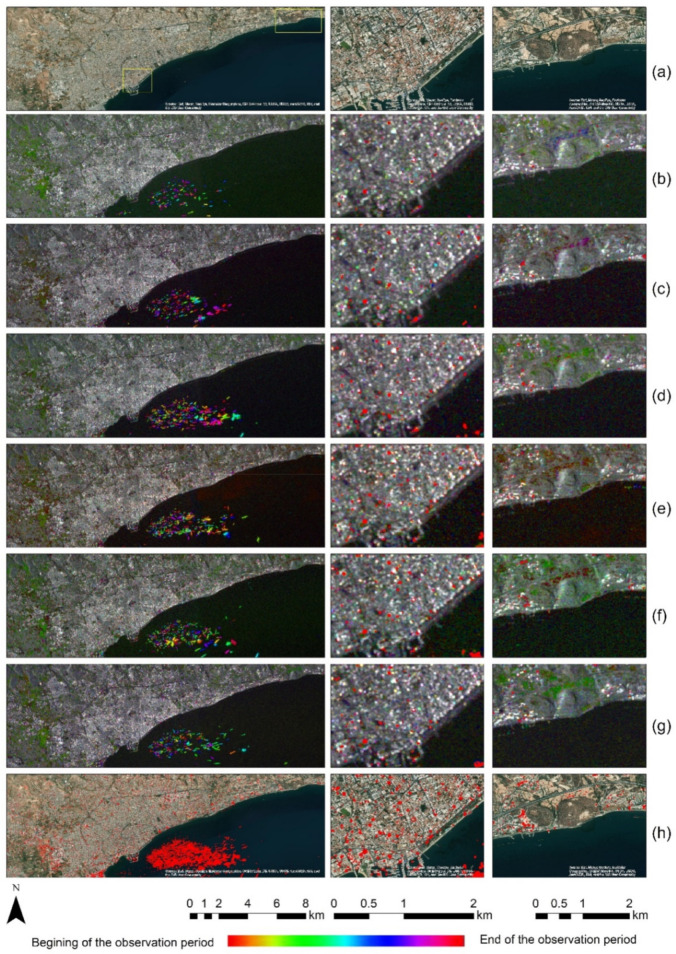
(**a**) High-resolution optical satellite image over the broader area of Limassol (left), city centre of Limassol (middle) and the Amathus archaeological site (right); (**b**) change detection analysis for the year 2015; (**c**) change detection analysis for the year 2016; (**d**) change detection analysis for the year 2017; (**e**) change detection analysis for the year 2018; (**f**) change detection analysis for the year 2019; and (**g**) change detection analysis for the year 2020. The cumulative changes for all years between 2015 and 2020 are shown in (**h**). At the bottom of the figure, a colour ramp indicates the relation of the colour according to the observation period for each sub-figure.

To further investigate the multi-temporal changes, over the area of interest, the REACTIV algorithm was implemented in the Google Earth Engine platform for each calendar year starting from the year 2015 until the year 2020. The overall results are depicted in Figure 5. A high-resolution optical satellite image over the broader area of Limassol, and closer looks over the centre of the city and the Amathus archaeological site are shown in Figure 5a. Figure 5 shows the change detection analysis for the year 2015; Figure 5c shows the results for the year 2016; Figure 5d shows the changes observed in the year after, while Figure 5e shows the changes for the year 2018. Finally, Figure 5f,g show the results for the years 2019 and 2020, respectively. Multi-temporal changes are highlighted with red colour over the city centre of Limassol (Figure 5 middle column) and the Amathus archaeological site (Figure 5 right column). The cumulative changes for all years between 2015 and 2020 are shown in Figure 5h.

Pixels that are highlighted with a red colour from Figure 5b to Figure 5g indicate changes observed at the beginning of each year (i.e., 2015, 2016 etc.), while pixels with purple colour indicate areas that have changed at the end of each observation year. Similarly, red colour pixels in Figure 5h indicate changes at the beginning of the observation period (2015) and purple colour changes at the end of this period (2020).

This time-span analysis provides supplementary information concerning the temporal changes in the broader area of the Limassol city, and visualises the spatial pattern of the changes. Changes are observed near the Old Port of Limassol (ships), in the urban fabric, as well as in the agricultural fields. In addition, changes in the urban fabric are detected along the coastline of the city (see also Section 4.1). A closer look at the land-use changes near the Amathus archaeological site can be seen in the right part of Figure 5 during the years 2015–2020 (with red colour).

Obviously, changes in the case of the Amathus archaeological site (right column of Figure 5) can be due to the phenological cycle of the agricultural fields (see also Section 5). These are mostly found in the north part of the site and indicated with a different colour in Figure 5b–e. This colour indicator is only related to observation time, and not with the type of change (see the colour ramp on the bottom of Figure 5).

For this study, we can assume that detection failures are those detections that are not linked with the urban expansion phenomenon. As already mentioned in [41], false positives can be driven due to two main reasons. Firstly, the high sensitivity of the response of agricultural areas can be seen in the case of the western part of the Limassol city (see changes in Figure 4h) and the previous example of the Amathus archaeological site, secondly during the so-called “point” events, which appear only on one date, as is the case of the ships located near the Old Port area. These detections could be further masked-out to minimise potential error. This analysis is also presented in the discussion section (Section 5).

### 4.3. Change Detection through Statistical Analysis

At the next step, a change detection statistical analysis was implemented based on the work of [42]. Here, the Google Earth Engine platform was used to apply a multi-temporal Wishart-based change detection during the period 2015–2020, using the Sentinel-1 radar images.

The outcomes for each year (i.e., 2015–2020) generate a new RGB image whereas the first component is referred as “cmap” which indicates the interval (time) of the most recent change. The second component is the so-called “smap” which indicates the interval of the first change, while the third component (“fmap”) shows the number of changes.

An example from this change detection technique can be seen in Table 2. This area is located approximately 700 m east of the Amathus archaeological site, on the seaside. In this area, a new large luxury hotel was successfully identified using the method mentioned earlier. Images from Google Earth (24 January 2015 and 5 April 2015) shows some preliminary earthworks in the specific parcel. The next available high-resolution image from Google Earth was on 24 April 2016, which indicates the construction phases of this hotel, while a more recent image taken on 12 September 2020, shows the final construction project. The last row of Table 2 shows on the left the results of the change detection method, using the Sentinel-1 datasets for the period 2015, while on the right part, the digitised results from this analysis are shown. Similar findings were retrieved for the same parcel for all other years of this study’s observation period (2015–2020). This time-stamp approach can help local stakeholders better understand the spatial–temporal urbanisation of the city.

**Figure 6:**
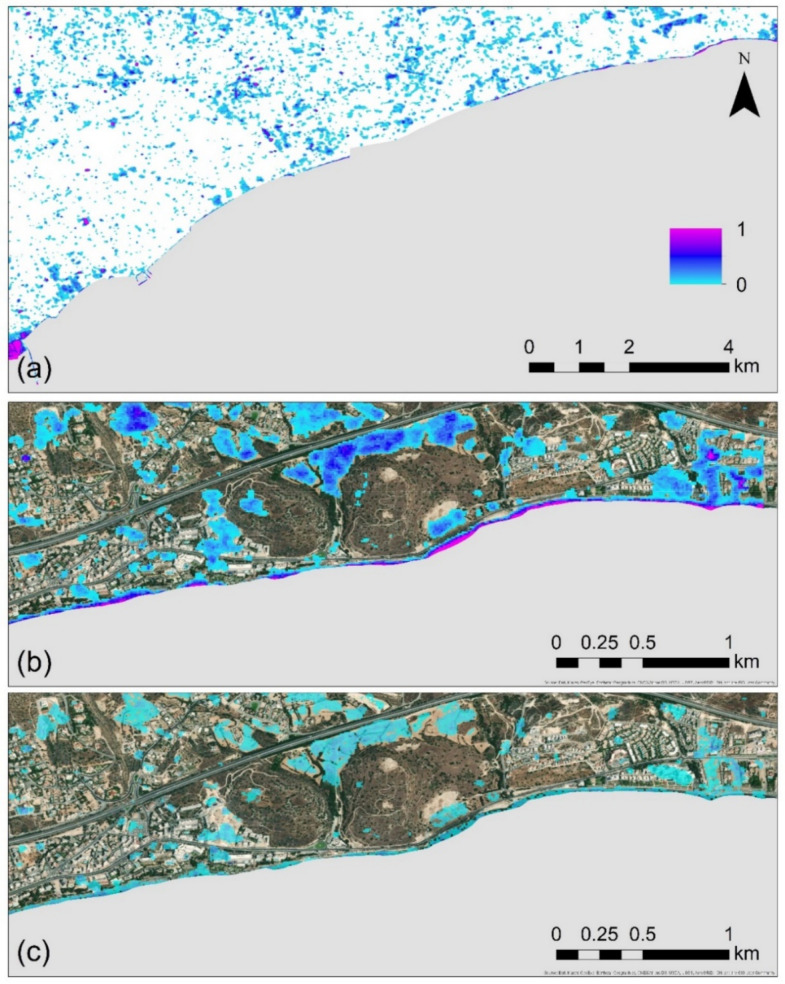
(**a**) Cumulative changes during the period 2015–2020 over the Limassol city, rescaled to 0–1, whereas zero (0) indicates areas with no changes and values close to one (1) area that have been changed in all Sentinel-1 images available in the Google Earth Engine; (**b**) a closer look at the cumulative changes during the period 2015–2020 around the Amathus archaeological site; and (**c**) the differences in relative changes between the year 2020 and 2015 around the Amathus archaeological site.

The “fmap” results for each year of observation (2015–2020) as mentioned earlier, indicate the number of changes. As this number might vary according to the number of the available Sentinel-1 images, a direct comparison of the changes between the different years is not possible. For this purpose, the “fmap” results were rescaled between 0 and 1. In contrast, the value 0 indicates areas with no changes and value 1 indicates areas with change for all images taken into consideration for the observation period. The overall results from this analysis are shown in Figure 6 below. Figure 6a indicates the cumulative rescale changes for the whole observation period (2015–2020) over Limassol. A mask was also applied for water bodies as these areas were susceptible to false alarms. Several areas, as shown in Figure 5a were highlighted as areas of changes. However, these changes are not directly linked with the urban sprawl phenomenon, but mostly to seasonal changes like agricultural fields and other non-built up areas (see Section 5, with a discussion on this false true).

An example of this is shown in Figure 6b, over the archaeological site of Amathus. Changes are observed in the northern part and on top of the hill of the archaeological site (acropolis). However, these changes are triggered due to the seasonal variations of the soil to vegetation and vice versa.

In contrast, land-use changes observed in the eastern and western parts of the site, are linked with construction projects during the period 2015–2020. Finally, Figure 6c indicates the differences between the reference years 2020 and 2015. The pattern is like those of Figure 5b.

## 5. Discussion

The previous results have highlighted various land-use changes in Limassol near the Amathus archaeological site. Of course, these changes might be a result of phenological changes in the agricultural fields of the city. In this study, we aimed to implement a quick, automatic, and low-cost exploration of large areas using medium-resolution free satellite datasets. To improve the overall results and overcome this critical point of false positives, a mask can be applied to overcome this.

Here, we generated a mask using a recent optical Sentinel-2 image (BOA, bottom of atmosphere-corrected images) through the Sentinel Toolboxes (SNAP). Using this recent multispectral Sentinel-2 image, a random forest classification was applied to detect urban areas. The supervised classification was carried out using training samples from the image, using various thematic classes (soil; vegetation; urban and water). The accuracy of the supervised classification was estimated to be more than 90%.

From this classification, the urban areas were isolated and used to mask out the results from Section 3. This allowed us to exclude areas that were not characterized as urban and therefore might be false positives results.

**Figure 7:**
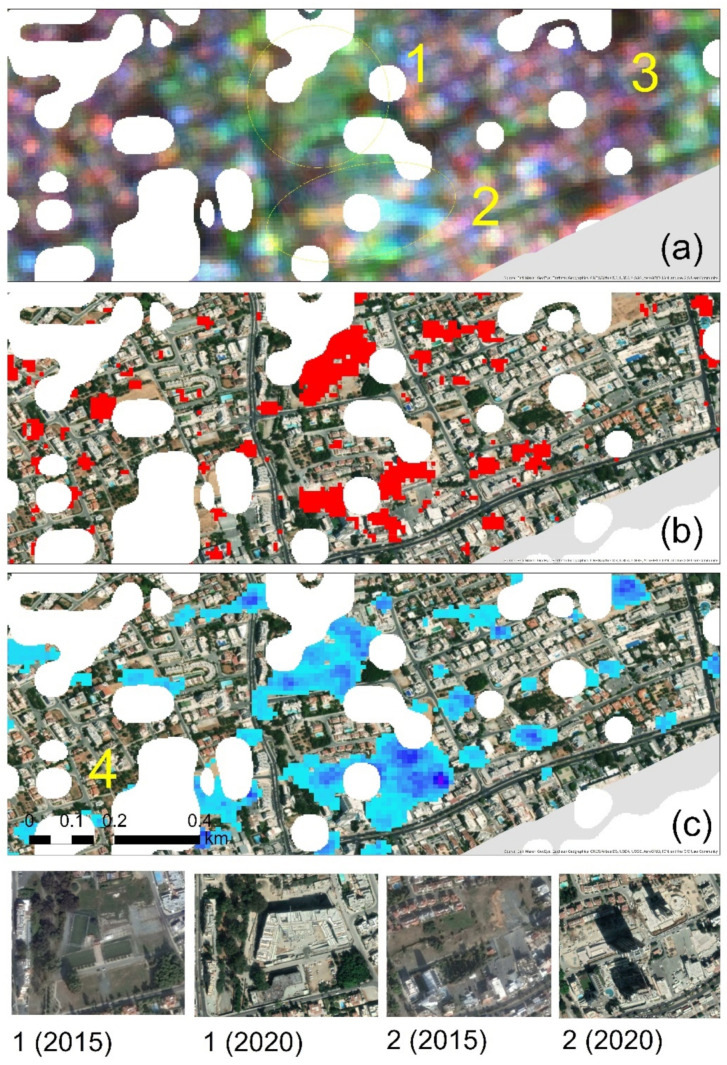
(**a**) VV and VH polarisations visualisation in Limassol city centre. The bright colour indicates changes within the observation period; (**b**) changes highlighted with red colour were recorded from the REACTIV algorithm for the same area as before; (**c**) cumulative changes using the statistical analysis. Specific areas mentioned in the text are shown with numbers 1–4. Non-urban areas, as classified from the optical Sentinel-2 image using the random forest classifier, are shown with white colour. On the bottom of the figure, screenshots of areas 1 and 2 were obtained from the Google Earth platform in 2015 and 2020.

A direct comparison of all the three methods mentioned above is difficult to be performed as they provide different outcomes. To overcome this problem and to carry out a cross-evaluation comparison between these methods, specific areas of interest have been selected, masked and presented in Figure 7. Figure 7a shows the VV and VH polarisations result over an area in the Limassol city centre. As already mentioned earlier, bright tones indicate areas of changes. Figure 7b shows for the same area the results from the HSV colour transformation after the implementation of the REACTIV algorithm, while Figure 7c shows the cumulative changes of the statistical change detection method as proposed by [42]. Non-urban areas, as classified from the random forest classification of the Sentinel-2 image, are shown with white colour. Screenshots from the Google Earth platform taken at the beginning of the observation period (2015) and by the end of this period (2020) are shown in the bottom of Figure 7. These screenshots correspond to the two areas (area 1 and area 2), indicated with a yellow circle in Figure 7a. For these two areas, all three methods tend to give similar results: high bright tones can be observed in both areas 1 and 2 in the VV and VH polarisations (Figure 7a). REACTIV results and the cumulative changes tend to give a similar pattern as well (Figure 7b,c), respectively.

However, in addition to areas 1 and 2, other dissimilarities can be detected between the three methods. For instance, another area with bright tones can be seen in the eastern part of the VV and VH polarisation composite (Figure 7a, area 3). This is partially also detected in Figure 7b and less in Figure 7c. This detection can be detected as a true false result for the current study’s needs, as in this area, no new construction or building was made during the observation period (2015–2020). The detected change should be linked to the use of this area as a parking place. On the western part of areas 1 and 2, in area 4 the cumulative change detection (Figure 7c) has detected some changes, in contrast to the rest of the methods. Based on the multi-temporal archives of the Google Earth platform, in this area, and during the observation period, two new constructions have taken place. Therefore, this detection was accurate for the cumulative change method, but this change could not be detected from the other two methods.

As it was shown, all three detection methods were able to detect the area’s major construction activities (areas 1 and 2). However, other smaller construction activities could not be detected by the REACTIV and the VV and VH polarisations. The cumulative method tends to provide the best results from these results, at least for this case study.

**Figure 8:**
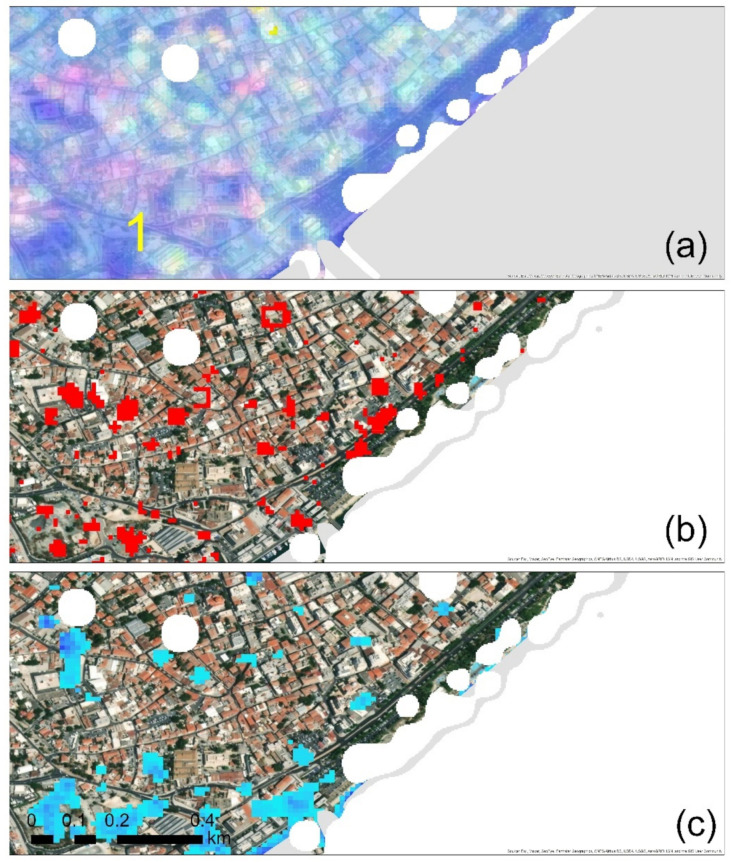
(**a**) VV and VH polarisations visualisation in Limassol centre. The bright colour indicates changes within the observation period; (**b**) changes highlighted with red colour as recorded from the REACTIV algorithm for the same area as before; (**c**) cumulative changes using the statistical analysis. Non-urban areas as classified from the optical Sentinel-2 image, using the random forest classifier, are shown with white colour. The specific area mentioned in the text is shown with numbering.

Similarly, an analysis at the centre of Limassol is shown in Figure 8. Figure 8a shows the results from the VV and VH polarisations, Figure 8b shows the REACTIV results and Figure 8c shows the results of the statistical analysis using the multi-temporal Sentinel-1 datasets during the period 2015–2020. Although all methods detected several changes in this area, only the one highlighted in Figure 8a (with the number 1) can be linked with the change in land-use. This change was recognised from all three different methods applied here. In the rest of the spotted areas, these are mostly linked with either true or false alarms (e.g., parking places).

## 6. Conclusions

Monitoring changes in the vicinity of archaeological sites and city centres are important information for local stakeholders. This study presented the results for the period 2015–2020, over the Limassol city in Cyprus. During this period, large construction projects have been initiated in various places within the city centre and the outskirts of the city. 

This study presented the results from the analysis of radar Sentinel-1 images, using the Google Earth Engine big data cloud platform. For the needs of the study, we implemented quick, automatic and low-cost exploration algorithms over large areas, for addressing this purpose. Three different methods have been implemented, starting from visualising the VV and the VH polarisations, then a colour space transformation (HSV) and finally, a statistical analysis of the Sentinel-1 images. 

While most of the detections from all methods were true, some were not linked with the phenomenon of urban sprawl and the construction projects, but rather to seasonal changes, agricultural practices, etc. This is considered as the main limitation of the aforementioned approach. To overcome this limitation, a “non-urban” mask was created based on a supervised classification analysis of a Sentinel-2 image. This mask can therefore mask out agricultural areas and other non-urban regions. A second critical limitation of the approach presented here refers to the spatial resolution of the Sentinel-1 radar images. The medium resolution of these images makes the detection of small changes in the urban fabric difficult.

These different methods should be further processed and integrated with other higher resolution data. This integration can further minimise the false alarms and maximise the accurate detections. The previous findings can be considered a first proxy indicator map for monitoring land-use changes and urban sprawl phenomenon, especially in areas with archaeological interest. Future work is expected to evaluate this quick approach for monitoring specific areas of interest (smaller areas) to identify any changes in the landscape and therefore support local stakeholders’ needs responsible for heritage management.

## Figures and Tables

**Table 1:** Examples of land-use changes (construction areas) thought the Google Earth satellite images, during the period 2015–2020 for the selected areas, as shown in Figure 3.

No	Before—2015 (24 January 2015)	After—2020 (12 September 2020)	No	Before—2015 (24 January 2015)	After—2020 (12 September 2020)
1	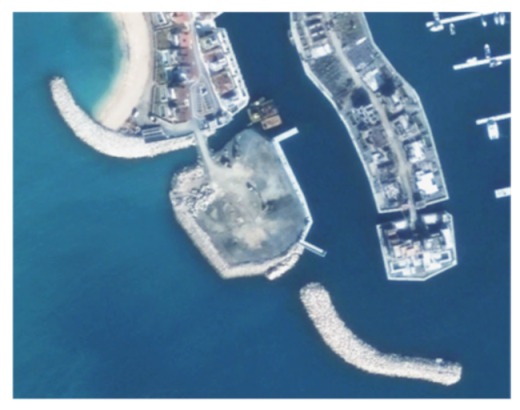	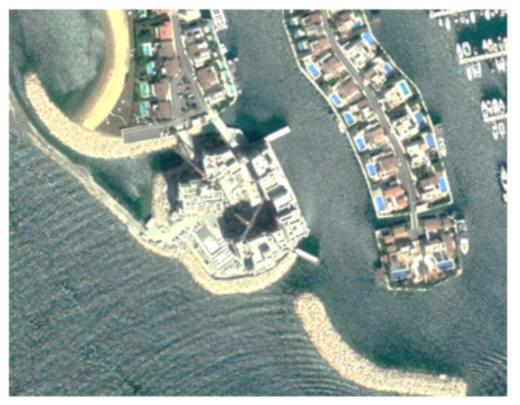	5	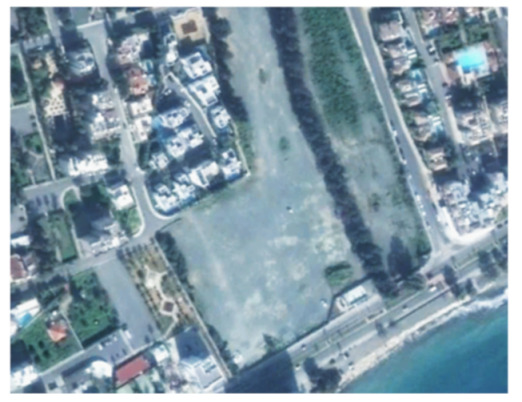	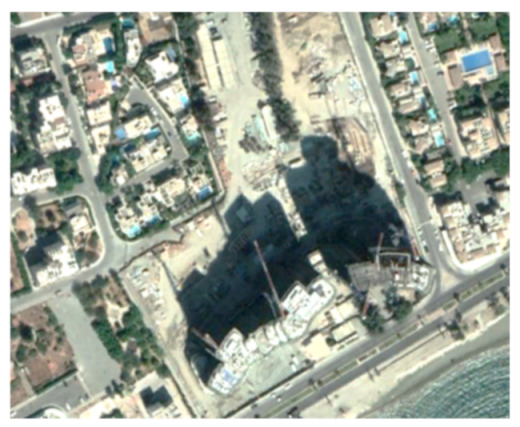
2	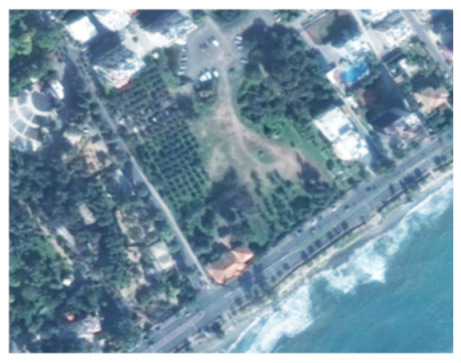	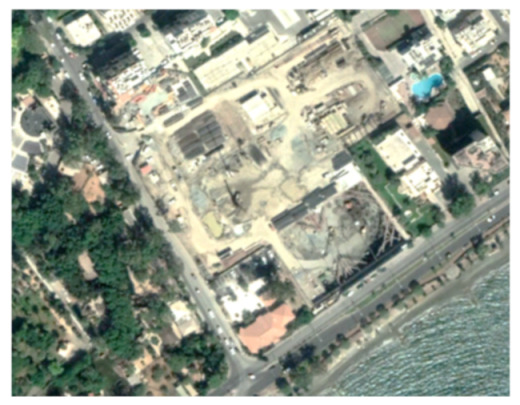	6	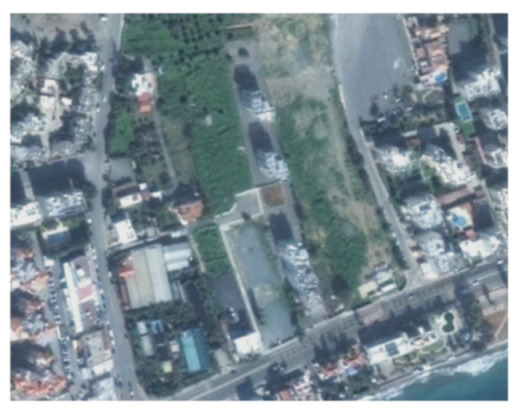	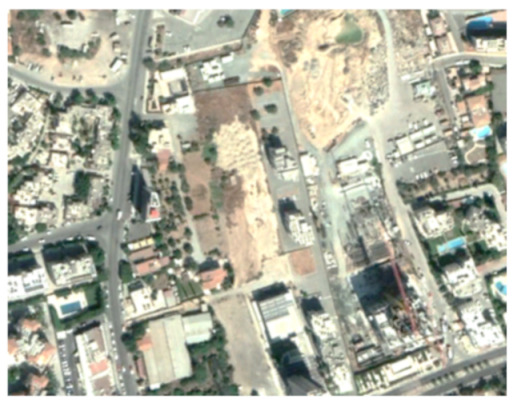
3	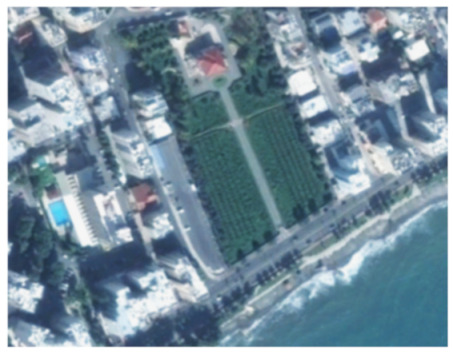	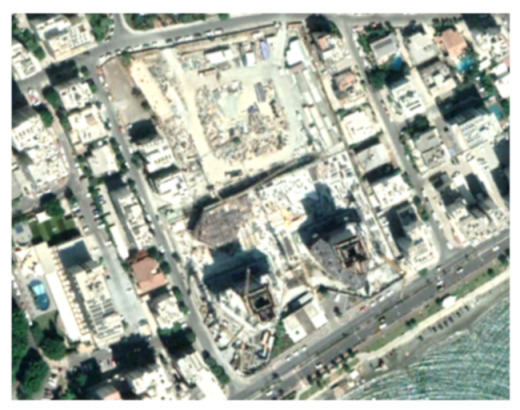	7	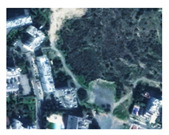	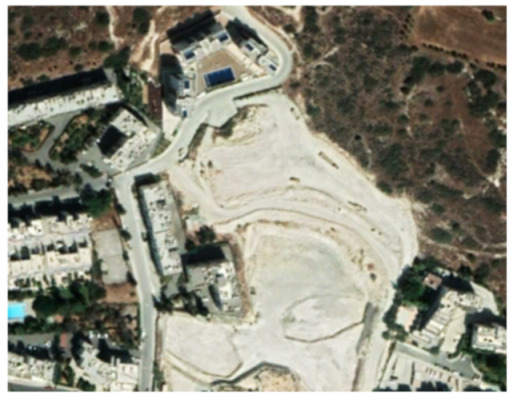
4	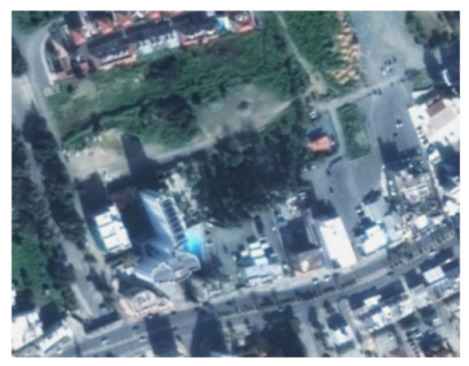	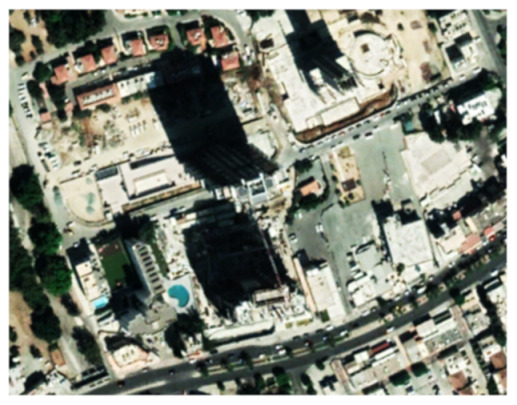	8	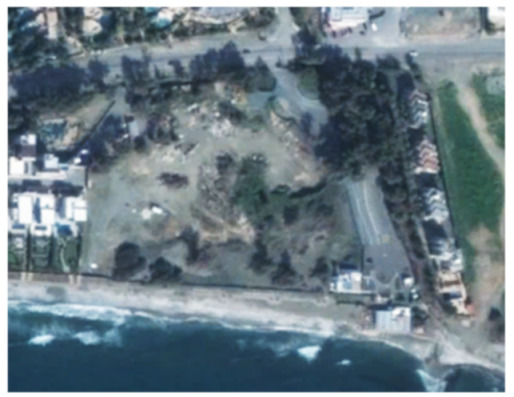	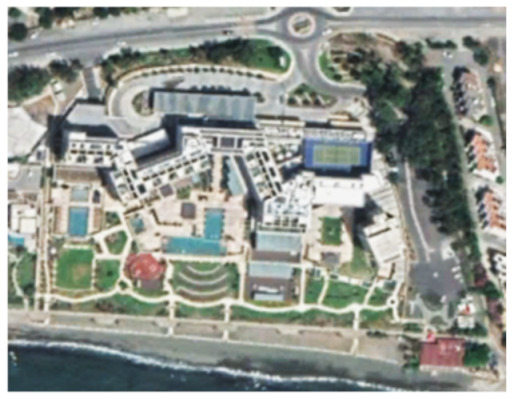

**Table 2:** Change detection results from just 700 m on the eastern part of the Amathus hill, using the 2015 Sentinel-1 images. A large construction was erected in the area.

Image (Google Earth)/Date	Image (Google Earth)/Date
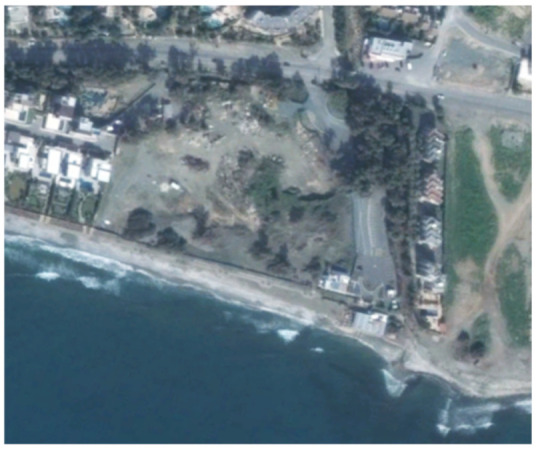	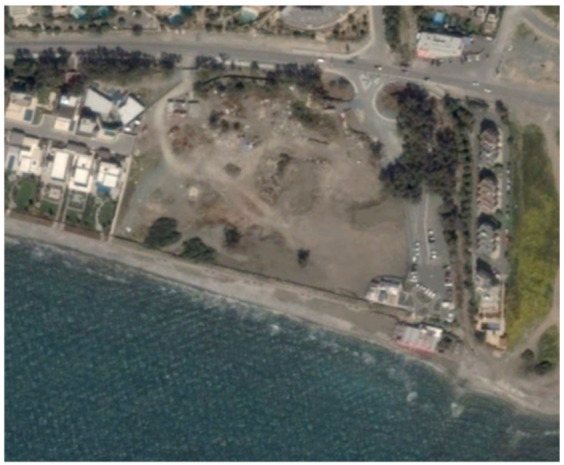
24 January 2015.	5 April 2015.
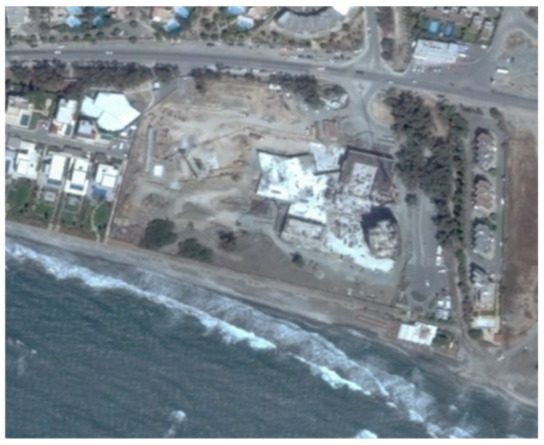	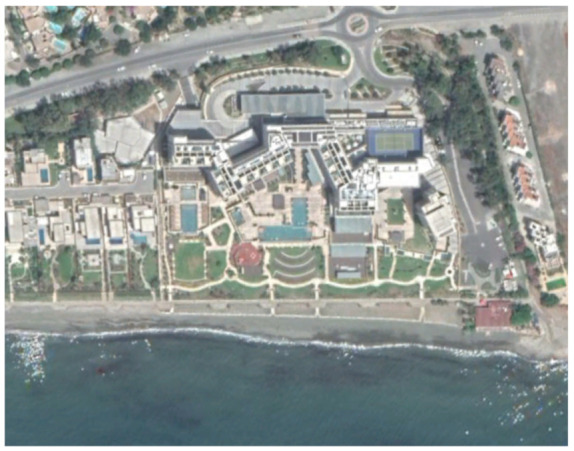
24 April 2016.	12 September 2020.
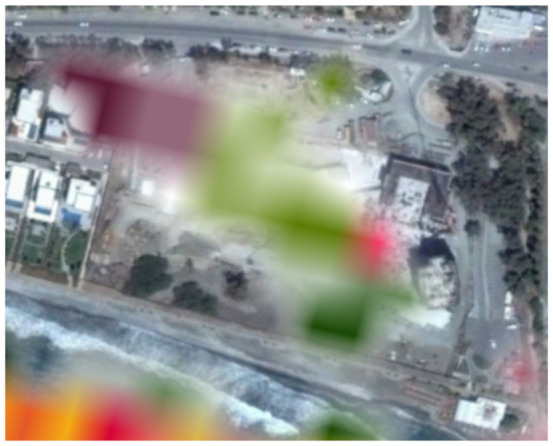	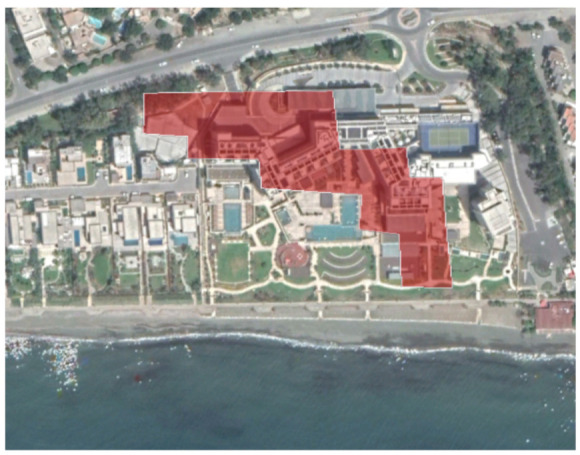
Change detection analysis using the Sentinel-1 2015 images.	Digitized change detection results (red polygon) of the Sentinel-1 2015 images, overlaid above the recent image of 12 September 2020.

## Data Availability

All available scripts used in this article can be found in the appropriated references, while the data used are freely accessible from the Sentinel Hub.
